# Do Chinese preschoolers follow adults’ suggestions? The impact of suggestion fairness and advisor familiarity on preschoolers’ sharing behavior

**DOI:** 10.3389/fpsyg.2025.1676175

**Published:** 2025-09-26

**Authors:** Chang Chen, Zihan Zha, Ru He, Ying Zhou, Wenjie Zhang

**Affiliations:** ^1^Faculty of Education and Science, Hunan Normal University, Changsha, China; ^2^Daxin Kindergarten, Shenzhen, China; ^3^Cognition and Human Behavior Key Laboratory, Hunan Normal University, Changsha, China; ^4^Institute of Psychology, Chinese Academy of Sciences, Beijing, China

**Keywords:** preschoolers, sharing behavior, suggestion fairness, advisor familiarity, emotional experience

## Abstract

**Introduction:**

While preschoolers’ sharing behavior is an important indicator of social development, it remains unclear how adult suggestions influence their sharing decisions.

**Methods:**

This study investigated how suggestion fairness (fair/unfair) and advisor familiarity (familiar/unfamiliar) affect preschoolers’ sharing behavior and its relationship with emotional experiences. Two experiments were conducted with 187 preschoolers aged 3-6 years (Experiment 1: *n* = 124, *M* = 4.54 years, *SD* = 1.02; Experiment 2: *n* = 63, *M* = 5.50 years, *SD* = 0.28) using a dictator game paradigm.

**Results:**

Results revealed that despite having a more mature understanding of fairness, 5-6-year-olds were more susceptible to adult suggestions compared to 3-4-year-olds. Older preschoolers were more likely to follow suggestions from familiar advisors while showing less compliance with unfamiliar advisors’ suggestions. Additionally, 5-6-year-olds demonstrated greater consistency between fairness judgments and actual sharing behavior, whereas 3-4-year-olds showed a larger cognition-behavior gap. Notably, children who shared more than they deemed fair (“over-sharing”) reported experiencing more positive emotions.

**Discussion:**

This study found that both suggestion fairness and advisor familiarity significantly influence preschoolers’ sharing decisions, with age-specific patterns in suggestion compliance and emotional experiences.

## Introduction

### Sharing behavior in preschoolers

Sharing behavior in early childhood represents a crucial milestone in social and emotional development that shapes children’s peer relationships, prosocial tendencies, and overall well-being (Brownell et al., 2013; Dunfield and Kuhlmeier, 2010). Recent research has highlighted that sharing behaviors emerge during the second year of life and continue to develop throughout the preschool years, with 4-year-olds demonstrating more spontaneous sharing compared to younger children (Fehr et al., 2008). The sharing concepts of 4 year-old children begin to gradually strengthen, but are still influenced by self-interest (Paulus, 2014), exhibiting certain “egocentricity.” The sharing behavior of 5–6-year-old children shows the most significant progress, with most of them being capable of “generous” sharing (Fehr et al., 2008). This may be influenced by social concepts, making 5–6-year-old children more eager to gain recognition from others, thereby demonstrating stronger altruistic motivation and moral concern. Thus, age 5 may represent a crucial turning point in the development of children’s sharing behavior.

There are multiple factors that influence sharing behavior in preschool children, among which children’s intrinsic understanding of fairness and equality is particularly crucial (Blake and McAuliffe, 2011). Fairness preference theory indicates that people not only focus on personal gains when making decisions, but also consider the fairness of outcome distribution schemes (Sen, 1997). Recent studies have revealed significant developments in fairness cognition among children aged 3–6 years, progressing from simple preferences for equal distribution to consideration of more complex fairness principles involving contribution and need (Blake et al., 2014). For instance, children aged 4–6 demonstrate heightened fairness sensitivity in resource allocation tasks, capable of identifying unfair distributions (Chernyak et al., 2019). However, a notable gap often exists between children’s fairness cognition and their actual sharing behavior, particularly during the preschool years. While 5–6-year-olds typically demonstrate mature understanding of fairness principles and can articulate what constitutes fair sharing, their actual sharing behavior may not consistently align with this understanding (Paulus et al., 2018). This “knowledge–behavior gap” appears particularly pronounced in younger preschoolers (3–4 years), who may verbally endorse equal sharing while still showing strong preferences for advantageous inequality in their actual behavior (Paulus, 2020). From the perspective of social information processing theory, preschoolers’ sharing behavior is not only influenced by internal fairness cognition, but also depends on the encoding, interpretation, and integration processes of external social information (Crick and Dodge, 1994). This theory emphasizes that social environments provide important cues for individuals to shape attitudes and behaviors, and individuals rely on this information to adjust themselves. Suggestions provided by others, as important social information input, significantly influence preschoolers’ cognitive processing and behavioral decision-making in sharing situations (Arsenio and Lemerise, 2004). External factors, such as suggestion fairness and advisor familiarity, all affect preschoolers’ sharing behavior performance through different information processing pathways.

Ages 3–6 represent a critical period for the development of preschoolers’ moral cognition and behavioral regulation abilities (Pushparatnam et al., 2021), and also constitute an important stage in the transition of sharing behavior from self-centered to socially-oriented. Current research faces a core controversy: are preschoolers’ sharing behaviors primarily driven by self-interest or influenced by external factors? Although existing studies have confirmed significant developmental trajectories in fairness cognition among 3–6-year-old children, the specific manifestations of the “knowledge–behavior gap” between this cognitive development and actual sharing behavior across different age groups remain unclear. This study focuses on 3–6-year-old preschoolers, combining fairness preference theory and social information processing theory, systematically exploring the developmental mechanisms of preschoolers’ sharing behavior from two dimensions: fairness cognitive level (internal) and others’ suggestions (external). The study aims to reveal the relationships between suggestion fairness and advisor familiarity with sharing behavior, and identify the critical age points where knowledge–behavior consistency emerges.

### Adult suggestions and fairness in children’s prosocial development

Beyond the internal development of fairness cognition, significant adults, such as parents, teachers, and caregivers, play a crucial role in shaping children’s social development, particularly their understanding of fairness and prosocial behaviors. Through interactions with these key figures, children acquire social norms and values, internalizing them as guiding principles for their own actions (Brownell et al., 2013).

A substantial body of research demonstrates the significant influence of adult suggestions and guidance on children’s prosocial behavior, including sharing. Adults can encourage sharing through verbal prompts or situational guidance, increasing both the frequency and amount of sharing (Paulus, 2018). Furthermore, adult feedback and evaluation play a crucial moderating role. When children’s sharing behaviors are met with positive feedback, they are more likely to continue exhibiting such behaviors in the future (Warneken and Tomasello, 2009).

The fairness of adult suggestions particularly impacts children’s prosocial behavioral development. When adults suggest fair sharing practices, such as equal distribution of toys among peers, it reinforces children’s understanding of fairness principles and promotes their implementation in real-world situations (Chernyak et al., 2019). Conversely, unfair suggestions, such as advocating that children keep most resources for themselves, may create confusion about fairness principles and potentially lead to the adoption of inequitable behavioral patterns (McAuliffe et al., 2020). Fairness preference theory suggests that individuals possess intrinsic fairness preferences and experience negative utility from unfair distributions, but these internal cognitive preferences are still in the developmental stage during the preschool period and are susceptible to influence and regulation by external social information. Current research lacks in-depth exploration of how fairness preferences are influenced by others’ suggestions, and how this influence interacts with fairness cognitive development in 3–6-year-old preschoolers remains unknown. Based on fairness preference theory, this study systematically examines the mechanisms through which suggestion fairness affects 3–6-year-old preschoolers’ sharing behavior, addressing the following core questions: Can suggestion fairness activate preschoolers’ intrinsic fairness preferences to promote sharing behavior, and does this activation effect exhibit age differences? Therefore, this study proposes the hypothesis: Compared to children who receive unfair suggestions, children who receive fair suggestions will demonstrate more generous sharing behavior (H1).

### Advisor familiarity in children’s prosocial development

Beyond fairness, the relationship between advisors and children, particularly advisor familiarity, significantly influences children’s behavioral development. Attachment research suggests that securely attached children are more likely to exhibit prosocial behaviors, such as sharing and cooperation (Thompson, 2016). This may be attributed to secure attachment providing a safe foundation for exploring social relationships and facilitating the understanding and adherence to social norms.

Familiar teachers, serving as crucial socialization agents, establish trust and attachment through daily interactions, making their suggestions more authoritative and influential on children’s behavior (Bretherton, 1992). Previously, it has been noted that teachers’ positive feedback on prosocial behavior can encourage children to exhibit more prosocial tendencies in subsequent situations, indicating that teachers subtly influence the development of preschoolers’ prosocial behavior (Ferreira et al., 2016). Research has shown that children tend to trust familiar individuals and view them as reliable and authoritative sources of information, thus being more inclined to follow their suggestions (Harris and Corriveau, 2011).

Currently, research on whether children’s sharing behavior differs based on suggestions from familiar versus unfamiliar advisors remains unverified. Based on social information processing theory, this study systematically examines the interactive effects of advisor familiarity and suggestion fairness on 3–6-year-old preschoolers’ sharing behavior, addressing the following core questions: Does advisor familiarity amplify the influence effect of suggestion fairness? Do these phenomena exhibit differences across different age groups? Therefore, this study proposes the hypothesis: Compared to unfamiliar advisors, children will demonstrate greater compliance with suggestions from familiar advisors, regardless of suggestion type (H2).

### Current study

Previous research has explored the developmental trajectory of sharing behavior and its associated factors, revealing that young children’s sharing is not entirely altruistic but influenced by self-interest and social relations, including recipient characteristics such as race, group membership, and social closeness. However, the impact of adult suggestions on preschoolers’ sharing behavior, particularly the interplay between suggestion fairness and advisor familiarity, remains under-investigated.

The present study aims to investigate how suggestion type (fair/unfair) and advisor familiarity (familiar/unfamiliar) influence sharing behavior in 3–6-year-old children. Additionally, we explore the relationship between children’s sharing behavior, their fairness judgments, and their emotional experiences. Through two experiments, we examine these relationships systematically.

Based on previous findings, we propose the following hypotheses:

*H1*: Children who receive fair suggestions will demonstrate more generous sharing behavior compared to those receiving unfair suggestions.

*H2*: Children will show greater compliance with suggestions from familiar advisors compared to unfamiliar advisors, regardless of the suggestion type.

*H3*: In the fair advice condition, children show more generous sharing behavior when advice comes from a familiar advisor compared to an unfamiliar advisor. In the unfair advice condition, children show less sharing behavior following advice from a familiar advisor compared to an unfamiliar advisor.

## Experiment 1: the influence of fairness suggestions and age on sharing behavior in preschool children

### Methods

#### Participants

A power analysis using G*Power 3.1 indicated that 90 participants would be sufficient to detect a medium effect size of *f* = 0.3 with a power of 0.80 and an alpha level of 0.05 (Faul et al., 2009). To ensure robust findings, we recruited 124 preschool children (aged 3–6 years) from a kindergarten in an urban area of central China. They comprised two age groups: 62 3–4 years (*M* = 3.55 years, *SD* = 0.30; 31 boys, 31 girls) and 62 five-to-six-year-olds (*M* = 5.52 years, *SD* = 0.28; 31 boys, 31 girls). All children had normal or corrected-to-normal vision, no history of mental or neurological disorders, and spoke Mandarin Chinese as their first language. The study was approved by the ethics committee, and written informed consent was obtained from all parents.

#### Experimental design

We employed a 2 (Age: 3–4 years, 5–6 years) × 2 (Suggestion fairness: fair, unfair) between-subjects factorial design. The independent variables were age group and suggestion fairness, and the dependent variable was the number of chocolates shared with an unfamiliar, same-gender recipient.

### Materials

#### Chocolates for the sharing task

Chocolates were selected as the sharing resource due to their popularity among preschoolers. Prior to the experiment, participants rated their preference for the chocolates using a three-point emoticon scale (“like,” “neither like nor dislike,” “dislike”) to ensure the sharing resource was appealing to them (Birch and Billman, 1986).

#### Photos of recipients

Two standardized photos of unfamiliar children (one boy and one girl) from a different kindergarten were used as recipients in the sharing task. Each photo measured 2.5 × 3.5 cm and depicted a headshot with a neutral facial expression. To control for potential gender effects, the gender of the recipient in the photo was matched to that of the participant.

#### Emotion scale

**Figure 1:**
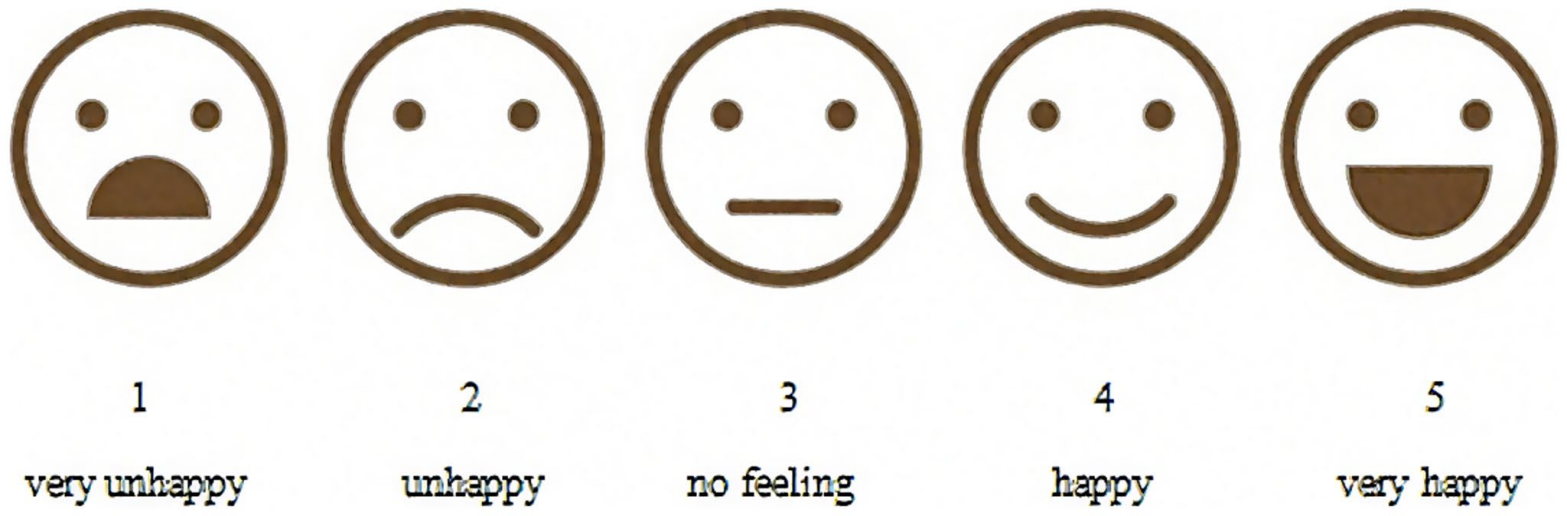
Five-point emotion scale.

To measure children’s emotional responses following the sharing task, a five-point emotion scale was used. The scale ranged from 1 to 5, representing emotions from negative to positive, using emoticons that varied from a crying face to a laughing face. This scale was a modified version based on the experiment by Ruggeri et al. (2017) (Figure 1).

#### Fair judgment card

**Figure 2:**
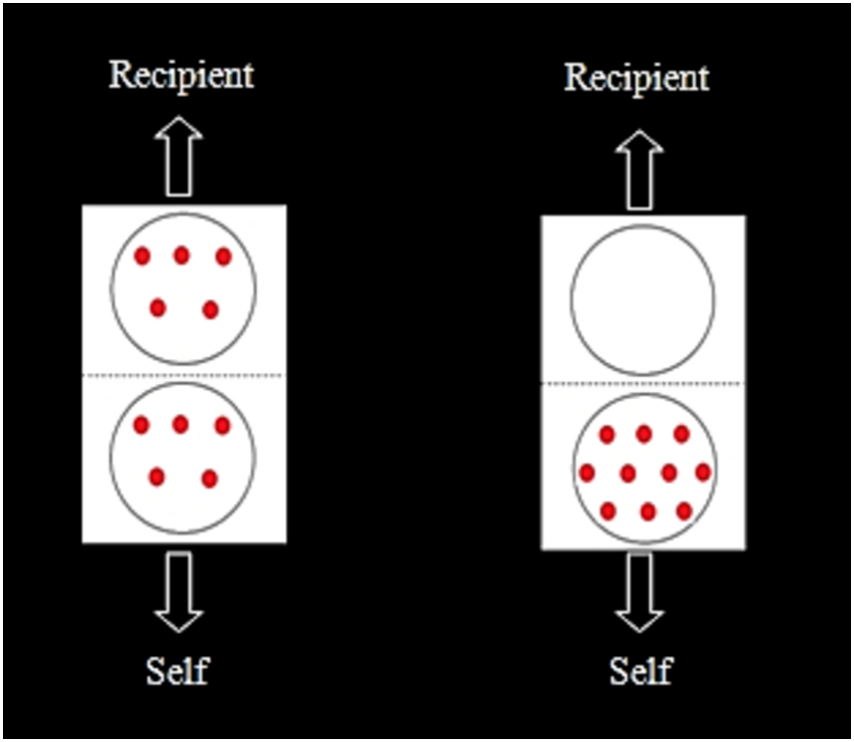
Fair judgement card.

A card illustrating fair and unfair chocolate distribution scenarios was used to assess preschoolers’ fairness judgment. The card displayed two options: a fair option where 10 chocolate beans were equally divided between the participant and the recipient (5/5), and an unfair option where all 10 chocolate beans were allocated to the participant and none to the recipient (10/0). Participants were asked to judge which option they considered fair (Figure 2).

### Procedure

#### Sharing resource preference rate and random condition assignment

The experiment was conducted in a quiet room within the kindergarten. The experimenter presented chocolates to each preschooler, who rated their preference using an emoticon scale (like, neither like nor dislike, dislike). Only those who liked the chocolates proceeded. Participants were then randomly assigned to either the fair or unfair suggestion group based on their student ID number: odd numbers to the fair suggestion group and even numbers to the unfair suggestion group.

#### Recipient introduction and suggestion manipulation

The experimenter presented a photo of an unfamiliar, gender-matched child as the recipient. In the fair suggestion group, participants heard from the experimenter, “If I were you, I would give half and keep half. I think it would be fair!” In the unfair suggestion group, they were told, “If I were you, I would keep them all; nobody will know!”

#### Sharing task

The experimenter then left the room briefly, leaving 10 chocolates on the desk. Participants decided how many chocolates to keep in their own bag and how many to place in a secret bag for the recipient.

#### Emotion report after sharing task

Upon completion of the sharing task, the experimenter returned to the room. Participants reported their emotions related to their sharing decision using a five-point emotion scale (very unhappy, unhappy, no feeling, happy, very happy).

#### Fair judgement test

**Figure 3:**
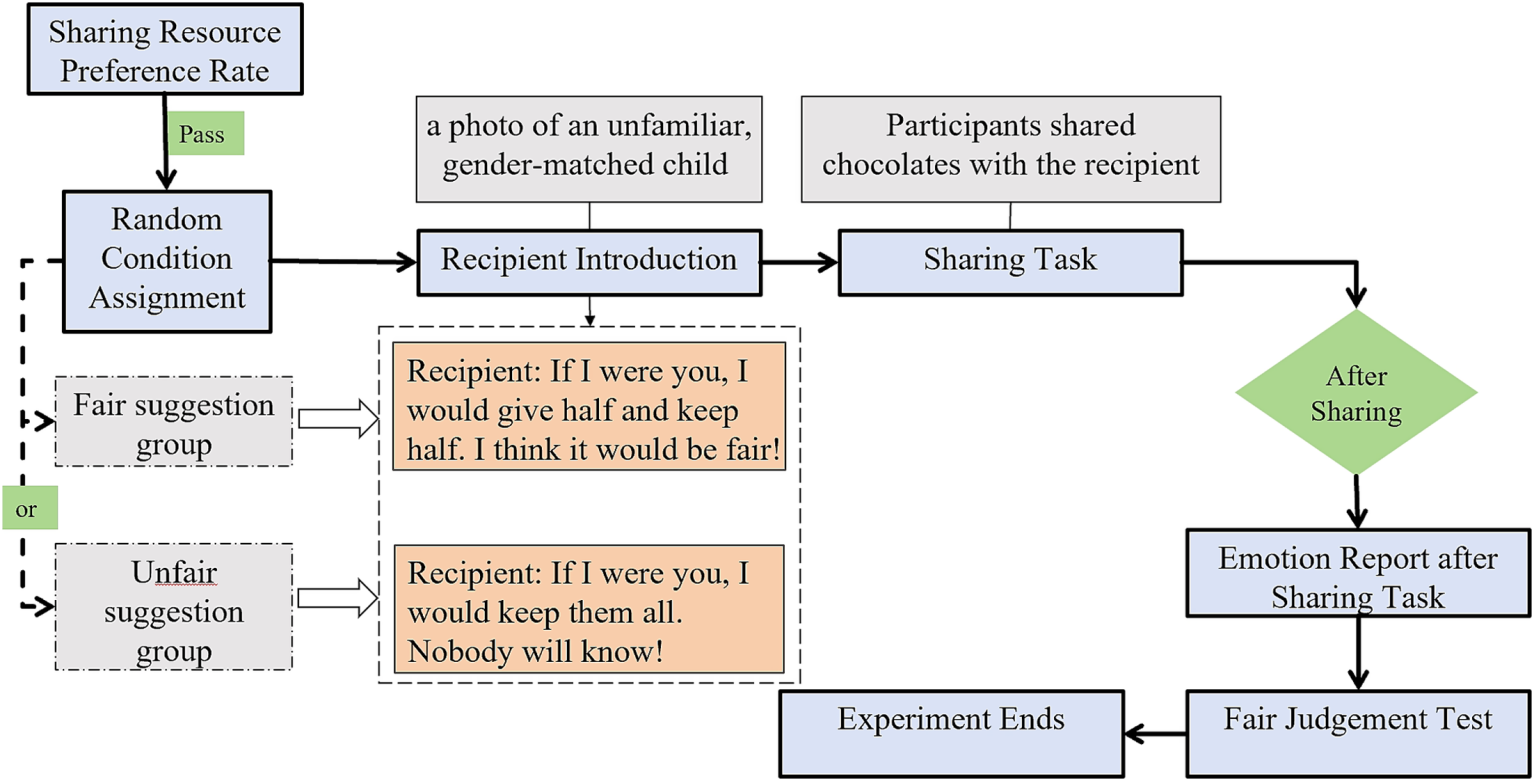
The procedure chart of Experiment 1.

Afterwards, the experimenter showed the fair judgment card to the participants, asking them to indicate which option they considered fair. The specific experimental procedure is shown in Figure 3.

### Data encoding and analysis

The number of chocolates shared by participants was recorded based on the quantity placed in the secret bag. For Experiment 1, a 2 (Age: 3–4 years, 5–6 years) × 2 (Suggestion fairness: fair, unfair) between-subjects ANOVA was conducted with the number of chocolates shared as the dependent variable. Fairness judgments were coded as 1 for equal distribution choices (5:5) and 0 for unequal choices (10:0). Dissonance was calculated as the difference between the actual sharing amount and the amount judged as fair, with positive values indicating sharing more than deemed fair (positive dissonance), zero indicating sharing the amount deemed fair (no dissonance), and negative values indicating sharing less than deemed fair (negative dissonance). A Spearman correlation was conducted to explore the relationship between dissonance and children’s emotional responses after the sharing task. All statistical analyses were performed using R software (Version 4.2.2). For significant interactions, we conducted *post hoc* analyses using Bonferroni-corrected *t* tests.

### Results

#### Number of chocolates shared

**Figure 4:**
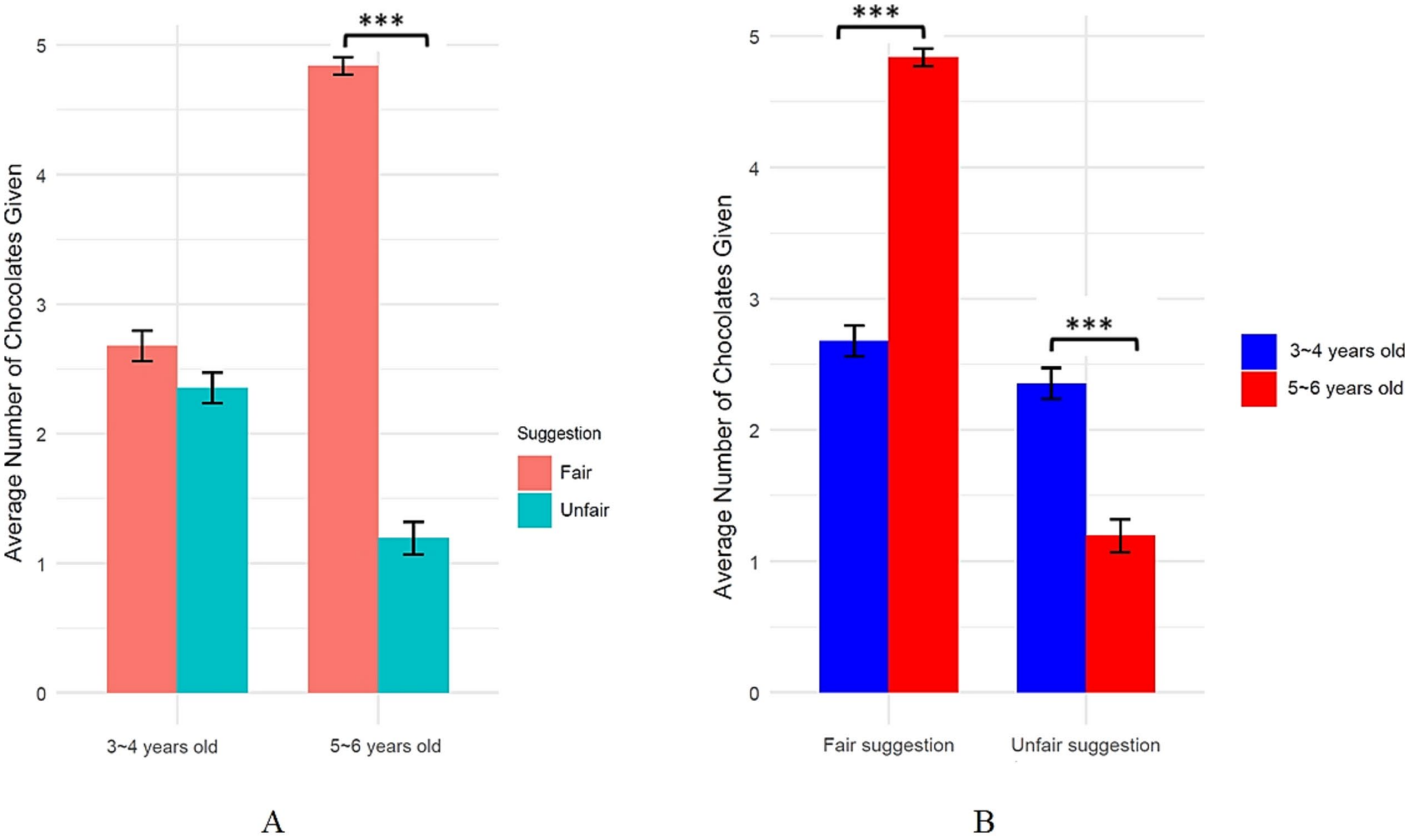
Differential effects of age **(A)** and suggestion types **(B)** in children’s sharing behavior at ages 3-4 and 5-6.

To examine the effects of suggestion fairness and age group on children’s sharing behavior, a two-way analysis of variance (ANOVA) was conducted. The analysis revealed a significant main effect of age group, *F* (1, 120) = 20.71, *p* < 0.001, *η*^2^ = 0.15, with older children (5–6 years) sharing more than younger children (3–4 years). There was also a significant main effect of suggestion fairness, *F* (1, 120) = 326.06, *p* < 0.001, *η*^2^ = 0.73, with more sharing in the fair suggestion condition than the unfair suggestion condition. Critically, there was a significant interaction between age group and suggestion fairness, *F* (1, 120) = 228.64, *p* < 0.001, *η*^2^ = 0.66. Simple effects analyses showed that while the suggestion fairness did not significantly affect sharing among 3–4 year olds, *p* = 0.058, it did for 5–6 year olds, *p* < 0.001. Specifically, among 5–6 year olds, those receiving the fair suggestion shared significantly more than those receiving the unfair suggestion. Further simple effects analyses revealed that in the fair suggestion condition, 5–6 year olds shared significantly more than 3–4 year olds, *p* < 0.001; whereas in the unfair suggestion condition, 5–6 year olds shared significantly less than 3–4 year olds, *p* < 0.001 (Figure 4).

#### Fair judgment, dissonance and emotion attribution

To examine age differences in fairness judgment, we compared the proportion of children who judged the 5:5 distribution as fair between age groups. Among children aged 3–4 years (*n* = 62), 51.61% judged the equal distribution (5:5) as fair, while this proportion increased to 95.16% among children aged 5–6 years (*n* = 62). Chi-square analysis revealed that children aged 5–6 years were significantly more likely to judge the equal distribution as fair compared to those aged 3–4 years, *χ*^2^(1, *N* = 124) = 27.91, *p* < 0.001. Furthermore, linear regression analysis indicated a significant positive correlation between age and children’s tendency to judge the equal distribution as fair, *b* = 0.017, *t* (122) = 6.05, *p* < 0.001, *R*^2^ = 0.231. This suggests that as age increased, children were more likely to consider equal distribution (5:5) as fair.

Dissonance was calculated as the difference between the actual sharing amount and the amount judged as fair, with positive values indicating sharing more than deemed fair (positive dissonance), zero indicating sharing the amount deemed fair (no dissonance), and negative values indicating sharing less than deemed fair (negative dissonance). Among children aged 3–4 years, 51.61% exhibited negative dissonance and 48.39% showed positive dissonance. For children aged 5–6 years, the distribution was markedly different: 50.00% demonstrated negative dissonance, 45.16% showed no dissonance (shared exactly what they judged as fair), and only 4.84% exhibited positive dissonance. Chi-square analysis revealed a significant association between age group and dissonance type, χ^2^ (2, *N* = 124) = 55.14, *p* < 0.001, suggesting that older children were more likely to demonstrate consistency between their fairness judgments and actual sharing behavior compared to younger children.

**Figure 5:**
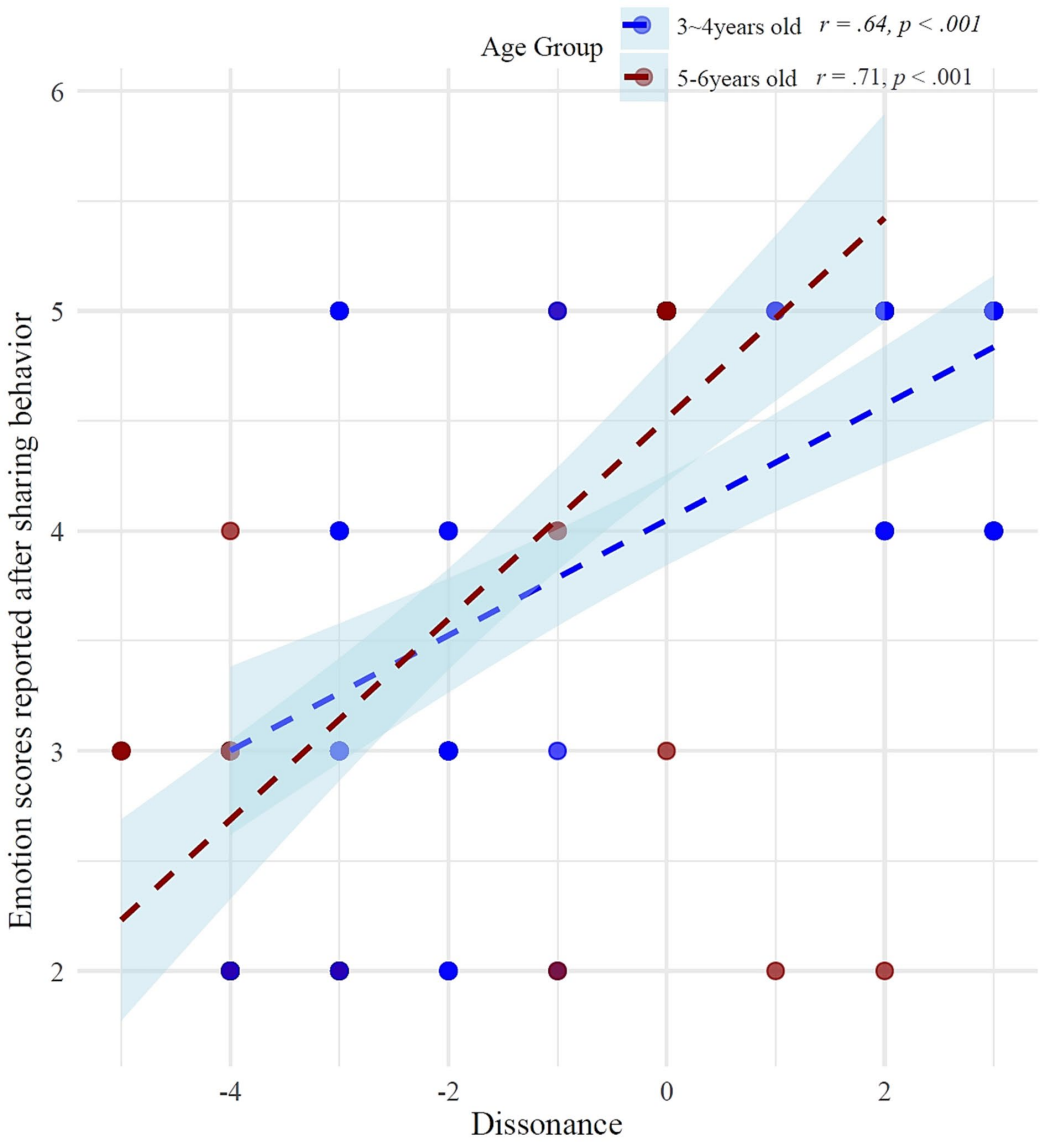
The correlation between dissonance (difference between actual sharing amount and the number deemed fair to share) and emotion scores reported after the sharing task.

Analysis of children’s emotional responses after the sharing task revealed significant correlations with behavioral-judgment dissonance (calculated as the difference between actual sharing amount and judged fair amount). Overall, there was a strong positive correlation between consistency and emotional response scores, *r* = 0.67, *p* < 0.001. Further age-specific analyses showed that this relationship was significant in both age groups, with a stronger correlation among children aged 5–6 years (*r* = 0.71, *p* < 0.001) compared to children aged 3–4 years (*r* = 0.64, *p* < 0.001) (Figure 5).

### Discussion

Experiment 1 revealed that 5–6-year-old preschoolers were significantly more responsive to the experimenter suggestions regarding sharing than their 3–4-year-old preschoolers. While the suggestion fairness did not significantly affect sharing among the younger children, 5–6-year-olds shared significantly more after receiving a fair suggestion and less after an unfair suggestion. This age-related difference in responsiveness to social suggestions aligns with previous research highlighting the developmental trajectory of generosity and prosocial behavior. Studies have shown a rapid increase in generosity around 5–6 years of age (Xiong et al., 2016) and a general trend of older preschoolers being more willing to share (Cowell and Decety, 2015). As children develop, they become less egocentric and more attuned to social cues and expectations (Lane et al., 2010). This increased social awareness likely contributes to the older children’s differentiated responses to fair and unfair suggestions. Furthermore, the greater impact of social models on older children (Fehr et al., 2008) may explain their heightened sensitivity to the experimenter suggestions in the sharing task.

The younger children’s lack of differentiation in sharing behavior across suggestion fairnesss may be attributed to their developing cognitive and social understanding. Three- and four-year-olds are still primarily egocentric, focused on their own immediate needs and desires (Zyryanova et al., 2016). Their limited theory of mind and executive functions (Devine and Hughes, 2014) may hinder their ability to fully grasp the implications of different suggestions and adjust their behavior accordingly. While they are beginning to transition from Piaget’s preoperational stage to the concrete operational stage, their understanding of social norms and perspectives is still nascent. This developmental stage, characterized by egocentrism, makes it challenging for younger children to integrate external suggestions into their decision-making processes, particularly when those suggestions conflict with their immediate preferences. In contrast, 5–6-year-olds demonstrate a growing capacity for social comparison and perspective-taking (Atance et al., 2010; Cigala and Mori, 2022; Gülay Ogelman et al., 2017), allowing them to better navigate social expectations and adjust their behavior in response to others’ influence. This developing understanding of fairness and social norms, enables older preschoolers to balance self-interest with social considerations, leading to more nuanced sharing behavior based on the perceived fairness of the suggestion.

Experiment 1 revealed that older preschoolers (5–6 years) were more susceptible to experimenter suggestions in their sharing behavior, despite their more mature understanding of fairness. However, the experimenter’s role as an advisor had certain limitations. Although the experiment was conducted in a quiet room within the preschool, the experimenter, being a special adult presence rather than a preschool teacher, might not fully capture children’s responses to adult suggestions in authentic educational settings. Moreover, the limited interaction and familiarity between the experimenter and children might have influenced children’s receptiveness to the suggestions.

Given the significant role of teachers as moral authorities in Chinese culture, examining the impact of teacher suggestions on children’s behavior becomes particularly important. To address the limitations of Experiment 1, we designed Experiment 2. By employing teachers as advisors, we aimed to create a more ecologically valid setting to investigate how teacher suggestions influence 5–6-year-old children’s sharing behavior. Specifically, Experiment 2 focused on examining how advisor familiarity (familiar vs. unfamiliar) and suggestion fairness (fair vs. unfair) jointly affect children’s sharing decisions.

## Experiment 2: the influence of fairness suggestions and advisor familiarity on sharing behavior in preschool children

### Method

#### Participants

A power analysis using G*Power 3.1 indicated that a minimum of 54 participants would be required to detect a medium effect size of *f* = 0.25 with a power of 0.95 and an alpha level of 0.05 (Faul et al., 2009). To ensure robust findings, we recruited 63 preschoolers aged 5–6 years (*M* = 5.50 years, *SD* = 0.28; 47.6% boys) from a kindergarten in an urban area of central China. All participants spoke Mandarin Chinese as their first language and had no reported history of mental or neurological disorders. The study was approved by the Ethics Committee and written informed consent was obtained from all parents prior to their children’s participation.

#### Experimental design

The experiment employed a 2 (Suggestion fairness: fair vs. unfair) × 2 (Advisor: familiar advisor vs. unfamiliar advisor) mixed design. Suggestion fairness served as a between-subjects factor, while Advisor was manipulated within subjects. The dependent variable was the number of chocolates shared by participants with an unfamiliar, same-gender the experimenter recipient.

### Material

The experimental materials in Experiment 2 were largely similar to those used in Experiment 1, with the following additions:

#### Inclusion of other in the Self Scale

**Figure 6:**
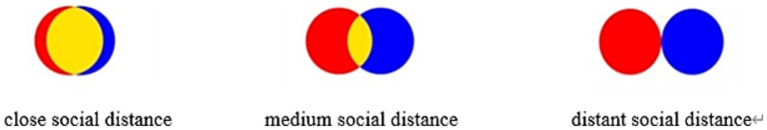
IOS Scale (early childhood version).

The Inclusion of Other in the Self Scale (IOS; Aron et al., 1992) was introduced in Experiment 2 to assess participants’ perceived social closeness with others. The IOS scale consisted of three pairs of circles with varying degrees of overlap, representing different levels of relationship closeness. Participants were informed that one circle represented themselves, while the other represented another person, with the degree of overlap indicating the closeness of their relationship, see Figure 6. Specifically, the first image represented a close relationship where the participant and the other person frequently interacted and played together. The second image indicated an acquaintance relationship with infrequent interaction. The last image denoted a distant relationship where the participant and the other person were unfamiliar and had no shared activities (Zhang et al., 2019). This scale was adapted to assess participants’ perceived closeness with their classroom teacher (familiar advisor) and a teacher from another kindergarten (unfamiliar advisor), see Figure 6.

#### Cartoon with audio for suggestion situation

**Figure 7:**
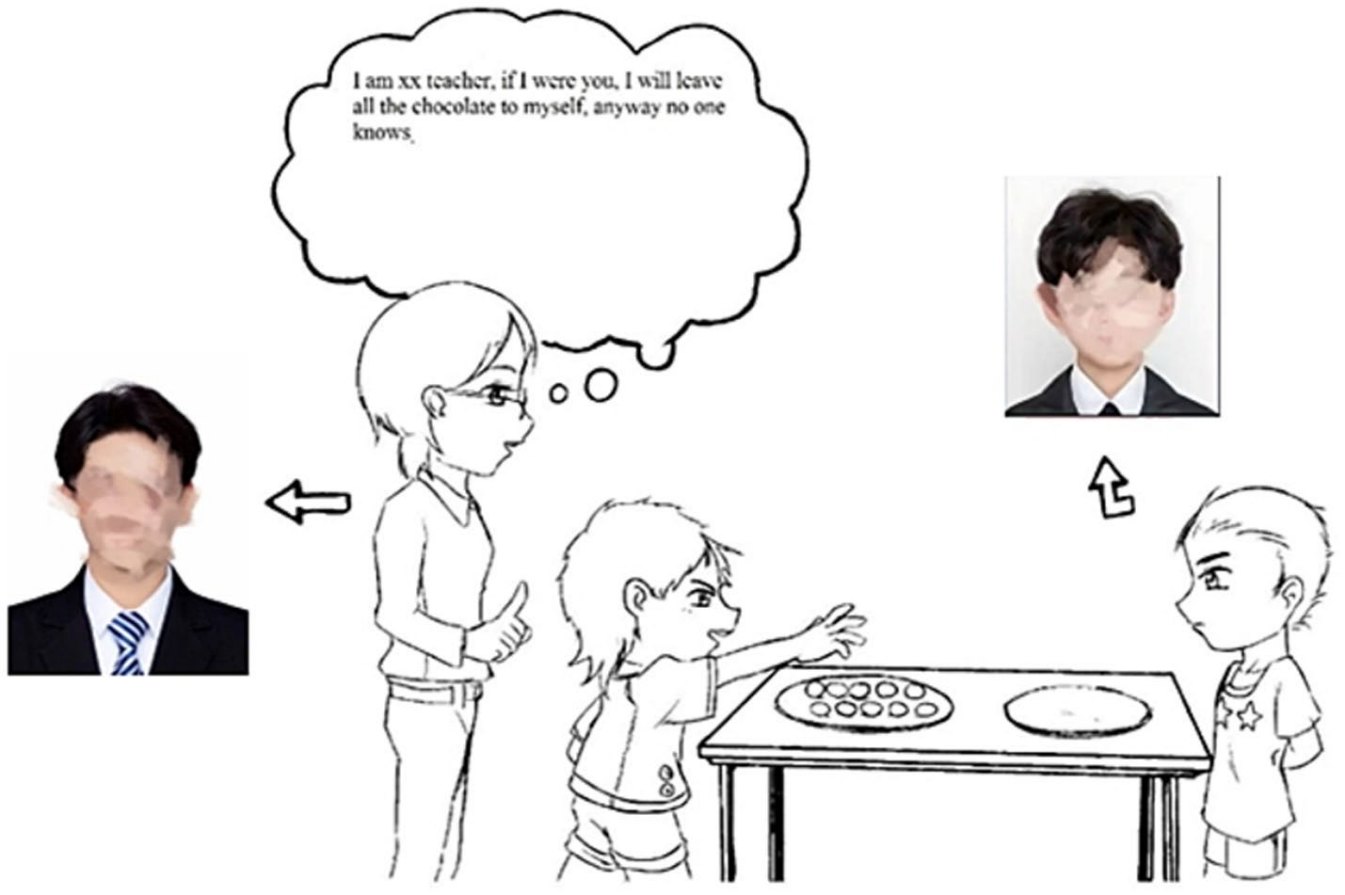
Cartoon of unfair suggestion situation.

To simulate the sharing scenario, a cartoon and corresponding audio recordings were developed. The cartoon visually depicted a sharing situation, featuring an advisor (either the fam 3–4 years iliar teacher or unfamiliar teacher) offering a suggestion while the recipient faced the participant. The cartoon provided a clear context for the sharing decision-making process. Accompanying the cartoon, pre-recorded audio clips were used to deliver the suggestions. For fair suggestions, the audio stated: “I’m [name] teacher. If I were you, I would give five chocolates and keep the other half for myself. I think this is fair.” For unfair suggestions, the audio stated: “I’m [name] teacher. If I were you, I would keep all the chocolates for myself, anyway no one knows.” These recordings were tailored to each advisor type (familiar advisor, unfamiliar advisor, or stranger) to ensure consistency with the visual representation in the cartoon (Figure 7).

### Procedure

**Figure 8:**
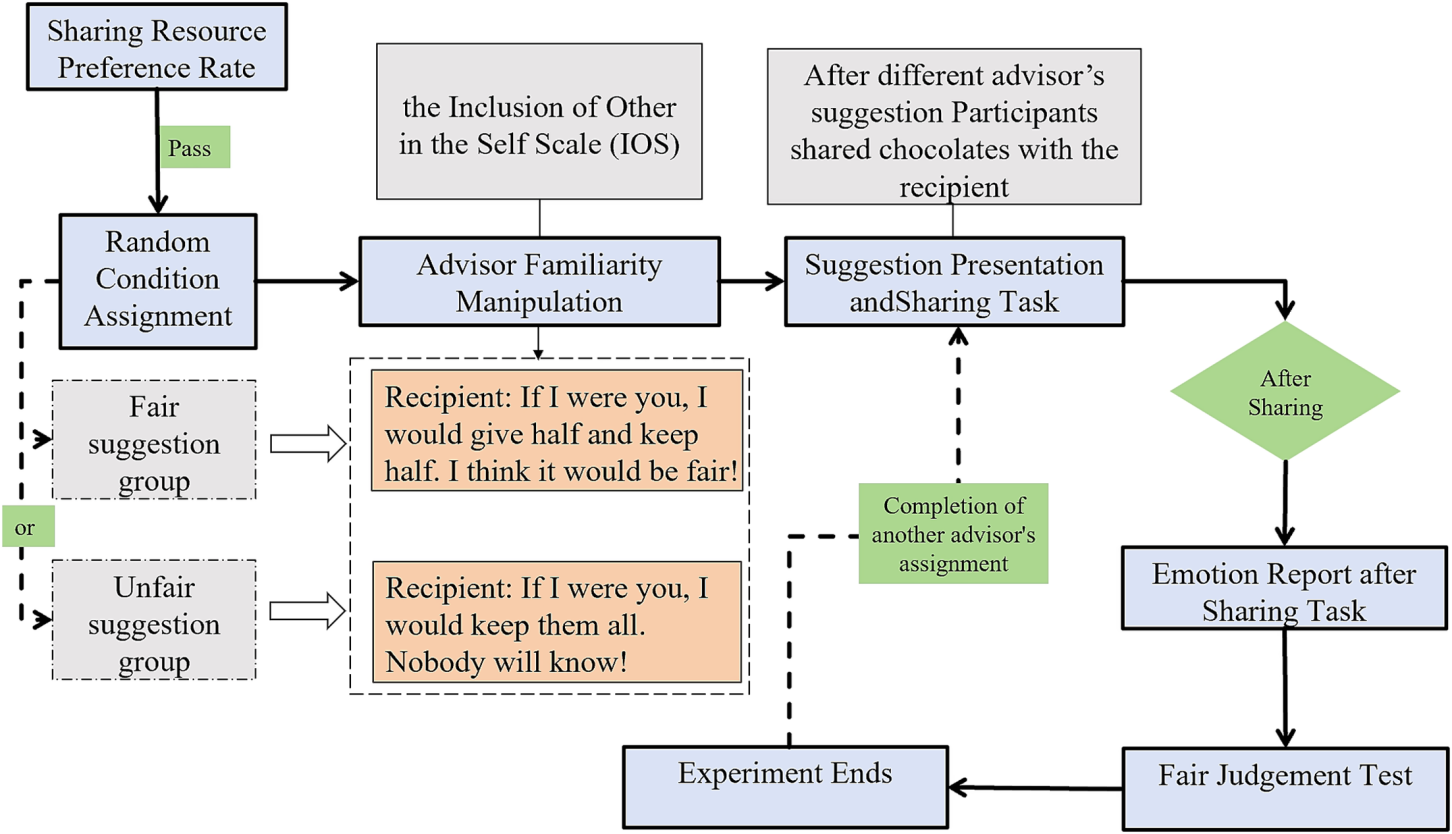
The procedure chart of Experiment 2.

Similar to Experiment 1, Experiment 2 began with participants rating their preference for chocolates using a three-point emoticon scale, with only those expressing preference proceeding to the study. Participants were then randomly assigned to either fair or unfair suggestion conditions based on their student ID numbers. The key modification in Experiment 2 was the introduction of advisor familiarity manipulation. Using the Inclusion of Other in the Self (IOS) scale (Aron et al., 1992), participants verified their perceived closeness with two advisors: their classroom teacher (familiar advisor) and a teacher from another kindergarten (unfamiliar advisor). As expected, all participants placed the familiar teacher at the highest closeness level (Level 1) and the unfamiliar teacher at the lowest level (Level 3) on the IOS scale, which displayed three pairs of overlapping circles representing varying degrees of relationship closeness (Zhang et al., 2019). The sharing task followed a similar format to Experiment 1, with participants viewing a cartoon depicting a sharing scenario while listening to an audio recording. However, in this experiment, either the familiar or unfamiliar advisor (randomized order) provided a suggestion aligned with the participant’s assigned condition. Following each sharing decision, participants completed the same emotion report (five-point scale from very unhappy to very happy) and fair judgment task as in Experiment 1. Notably, each participant completed two trials of the sharing task and subsequent measures, one with each advisor (familiar and unfamiliar advisor, order randomized) (Figure 8).

### Data encoding and analysis

Experiment 2 was analyzed using a 2 (suggested fairness: fair, unfair) × 2 (advisor: familiar, unfamiliar) mixed ANOVA. The number of chocolates shared was recorded. Fairness judgments were coded as 1 for equal distribution (5:5) and 0 for unequal distribution (10:0 or 0:10). Dissonance was calculated as the difference between the actual sharing amount and the amount judged as fair. Positive values indicated sharing more than deemed fair (positive dissonance), zero indicated sharing the amount deemed fair (no dissonance), and negative values indicated sharing less than deemed fair (negative dissonance). Spearman correlations were conducted to explore the relationship between dissonance and children’s emotional responses after the sharing task. All analyses were performed using R (Version 4.2.2). For significant interactions, Bonferroni-corrected *t*-tests were used for *post hoc* comparisons.

### Results

#### Number of chocolates shared

2 (Suggestion fairness: fair vs. unfair) × 2 (Advisor: familiar vs. unfamiliar) mixed ANOVA was conducted to examine the effects of suggestion fairness and advisor familiarity on children’s sharing behavior. The analysis revealed a significant main effect of suggestion fairness, *F* (1, 61) = 48.65, *p* < 0.001, *η*^2^ = 0.246, with children sharing more chocolates in the fair suggestion condition compared to the unfair suggestion condition. A significant main effect of advisor familiarity was also found, *F* (1, 61) = 20.16, *p* < 0.001, *η*^2^ = 0.163, indicating that children shared more chocolates when receiving suggestions from familiar advisors compared to unfamiliar advisors. Importantly, there was a significant interaction between suggestion fairness and advisor familiarity, *F* (1, 61) = 137.40, *p* < 0.001, *η*^2^ = 0.571.

**Figure 9:**
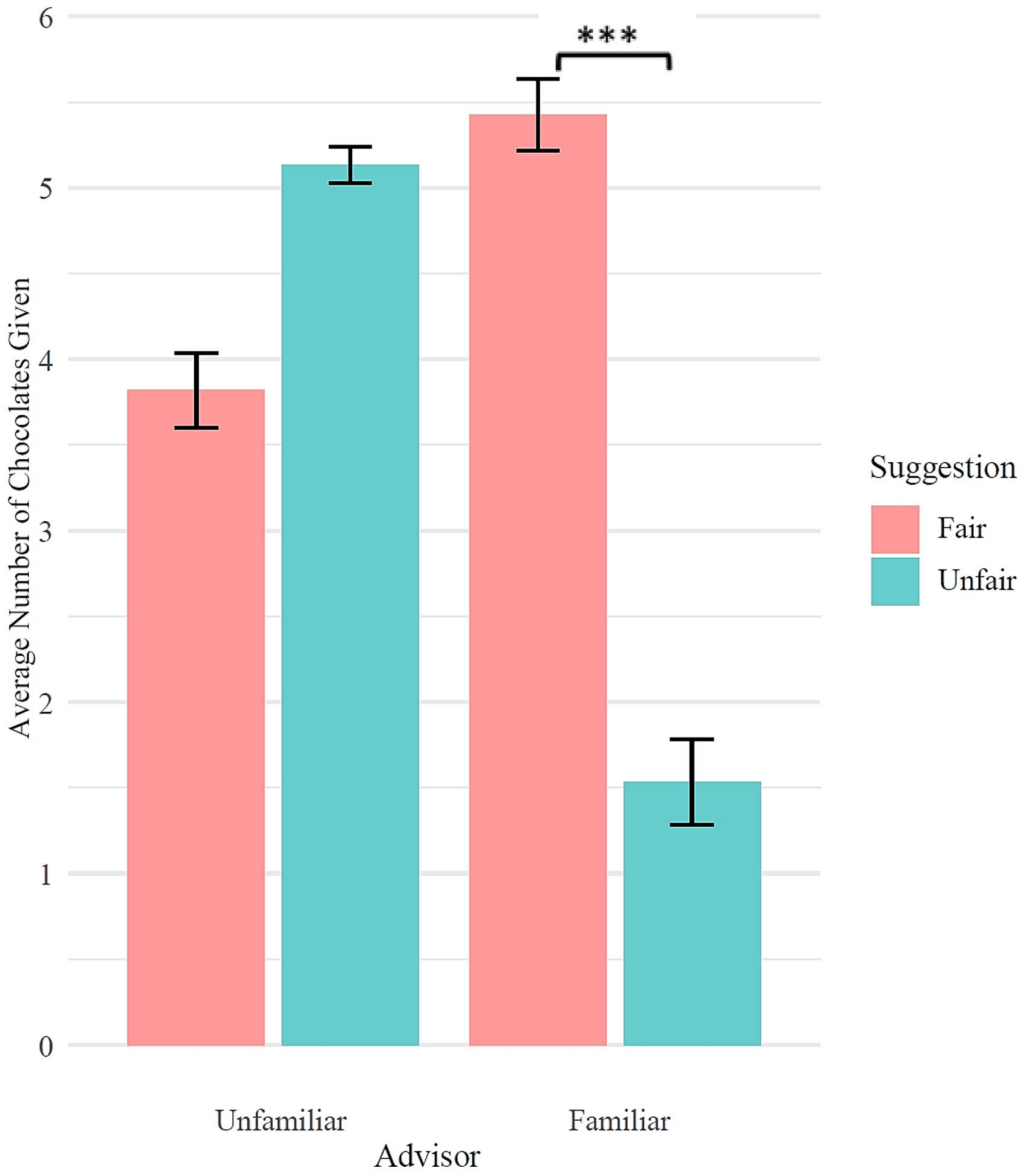
Effects of suggestion type from unfamiliar and familiar advisor on children’s sharing behavior.

Further simple effects analyses revealed that for the unfamiliar advisor, there was no significant difference in the number of chocolates shared between the fair (*M* = 3.82, *SD* = 1.26) and unfair (*M* = 5.13, *SD* = 0.57) suggestion conditions, *p* = 0.119. However, for the familiar advisor, children shared significantly more chocolates in the fair suggestion condition (*M* = 5.42, *SD* = 1.20) compared to the unfair suggestion condition (*M* = 1.53, *SD* = 1.63), *t* (120) = 7.49, *p* < 0.001 (Figure 9).

**Figure 10:**
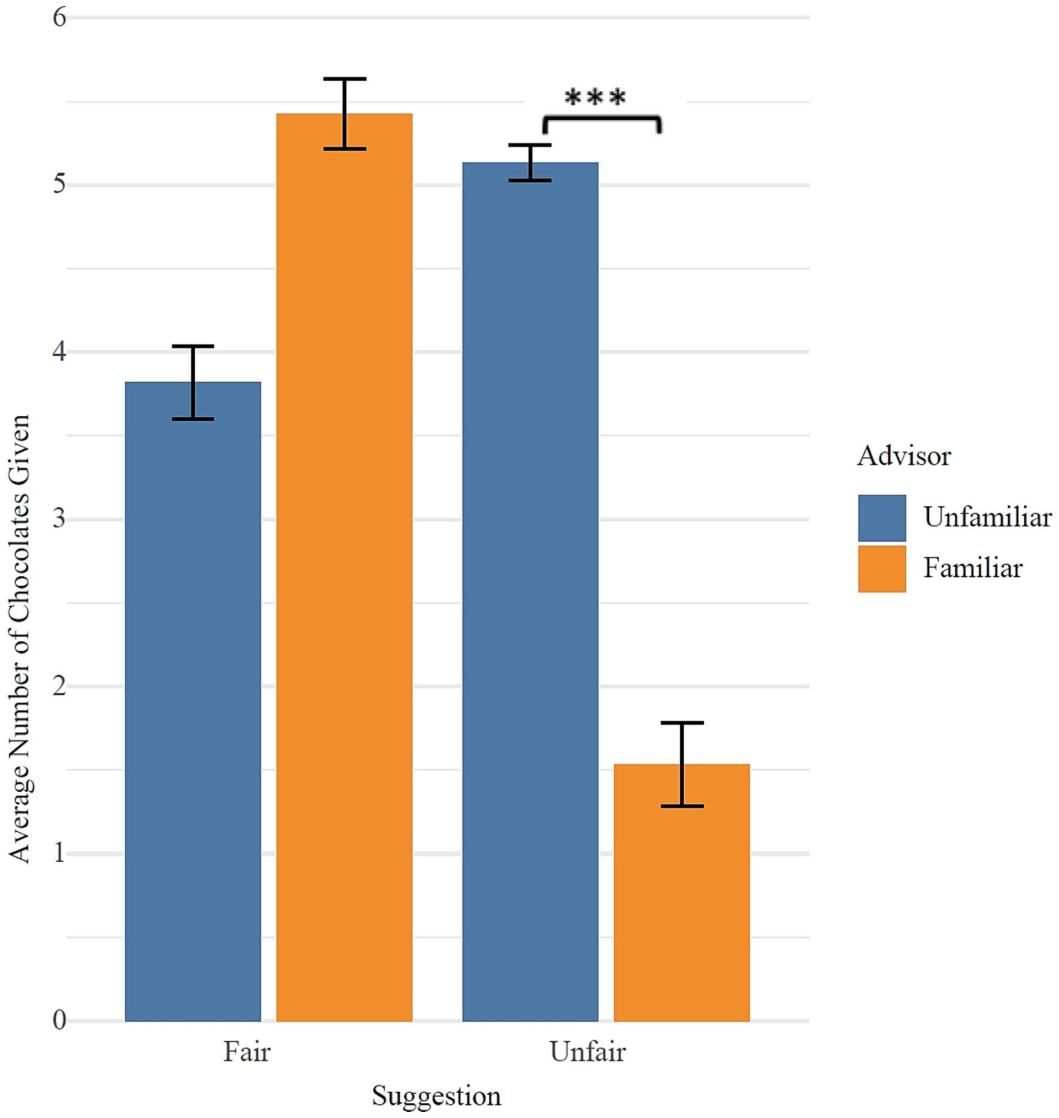
Effects of advisor familiarity from fair and unfair suggestion on children’s sharing behavior.

Furthermore, under the fair suggestion condition, children shared significantly more chocolates when the suggestion came from a familiar advisor (*M* = 5.42, *SD* = 1.20) compared to an unfamiliar advisor (*M* = 3.82, *SD* = 1.26), *t* (32) = −4.77, *p* < 0.001. Conversely, in the unfair suggestion condition, children shared significantly fewer chocolates after suggestions from a familiar advisor (*M* = 1.53, *SD* = 1.63) compared to an unfamiliar advisor (*M* = 5.13, *SD* = 0.57), *t* (29) = 12.80, *p* < 0.001 (Figure 10).

#### Fair judgment and emotion attribution

Consistent with Experiment 1, fairness judgments in Experiment 2 revealed that the vast majority of 5–6-year-olds (96.83%) considered an equal distribution (5:5) to be fair, while only a small minority considered a 0:10 (all chocolates kept) distribution to be fair.

Dissonance was calculated as the difference between children’s actual sharing amount and the amount they judged as fair. Consistent with Experiment 1, among 5- to 6-year-old children in Experiment 2, similar proportions exhibited no dissonance (46.8%, sharing the amount judged as fair) and negative dissonance (46.0%, sharing less than what they deemed fair). A considerably smaller proportion (7.14%) displayed positive dissonance (sharing more than what they considered fair).

In Experiment 1, dissonance was observed between participants’ moral judgments and their sharing behaviors, and this was associated with their emotional responses. The findings from Experiment 2 similarly revealed this dissonance, regardless of the advisor type providing the suggestion.

**Figure 11:**
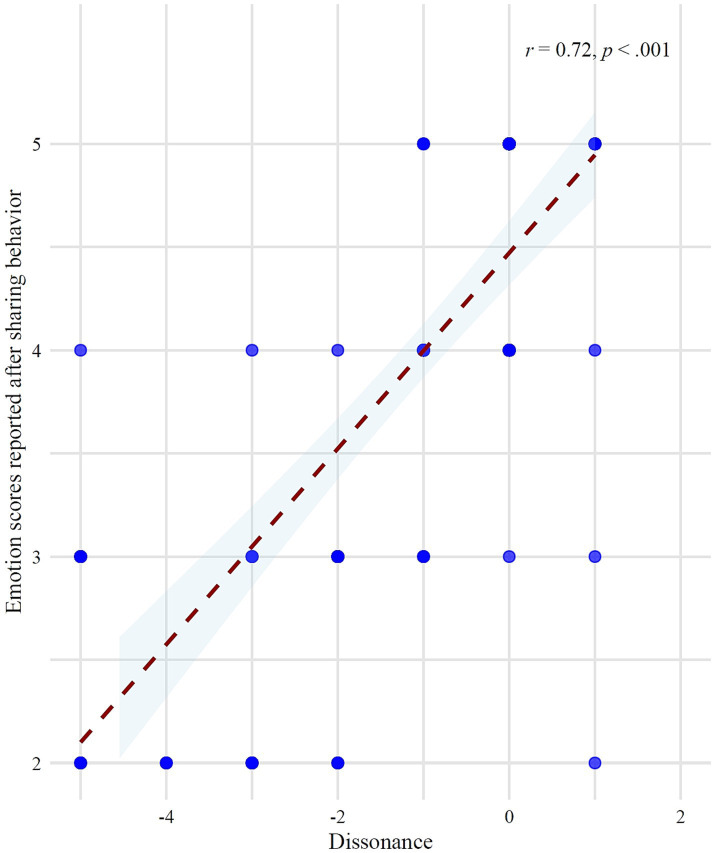
The correlation between dissonance (difference between actual sharing amount and the number deemed fair to share) and emotion scores reported after the sharing task.

To examine the relationship between behavioral-judgment dissonance and emotional responses, a Spearman rank correlation analysis was conducted. Consistent with Experiment 1, among 5- to 6-year-old children in Experiment 2, results revealed a strong positive correlation between behavioral-judgment dissonance and emotional responses (*r* = 0.72, *p* < 0.001) (Figure 11).

### Discussion

This study found that 5- to 6-year-old children’s receptiveness to suggestions was closely related to their familiarity with the advisor. When the suggestion came from a familiar advisors, children were more inclined to follow the suggestion and adjust their sharing behavior based on the fairness of the suggestion. This aligns with previous findings on children’s preference for trusting familiar individuals in social learning (Yu et al., 2016). Familiar advisors, serving as important socialization agents, establish trust and attachment through daily interactions (Ozmete, 2011), giving their suggestions greater influence on children’s behavior. Children often adjust their social behavior according to the familiarity of the builder (Rakoczy et al., 2015). Furthermore, suggestions from familiar advisors may elicit stronger social expectations, motivating children to adhere to the suggestions to gain approval and praise (Carter and Pool, 2012). Research indicates that children exhibit greater trust and dependence on familiar social members (Danovitch and Mills, 2014), and familiar social relationships enhance the credibility and influence of suggestions (Danovitch and Mills, 2014). Therefore, suggestions from familiar advisors are more likely to be perceived as authoritative and trustworthy, leading children to accept them unconditionally, regardless of whether the suggestions align with fairness principles.

In contrast, when faced with unfamiliar advisors, children lack a pre-existing foundation of trust and may harbor doubts about the reliability and validity of the suggestions. Social information processing theory posits that children consider the credibility and relevance of the source when processing social information (Crick and Dodge, 1994; Shtulman, 2024). Due to the absence of prior interaction and emotional connection with unfamiliar advisors, children may rely more on their own judgment to determine their sharing behavior. They are more likely to base their decisions on their own volition and judgment rather than blindly following the suggestions of unfamiliar individuals. Consequently, when suggestions come from strangers, children’s sharing behavior is more likely to reflect their own preferences than adherence to the suggestion.

## General discussion

### Developmental leap in fairness cognition: 5–6-year-old preschoolers’ superior understanding of equal distribution

Experiments 1 and 2 consistently demonstrated that 5–6-year-old preschoolers possess a more mature understanding of fairness, equating it with equal distribution. Experiment 1, while 51.61% of 3–4-year-olds judged a 5:5 distribution as fair, this proportion increased significantly to 95.16% in 5–6-year-olds. Experiment 2 corroborated this finding, with 96.83% of 5–6-year-olds identifying the 5:5 distribution as fairest. These results align with previous research indicating a developmental shift in fairness cognition around this age, where children increasingly associate fairness with equal resource allocation (Hardecker et al., 2016; Li et al., 2016; Rochat et al., 2009). The theory of fairness preference argues that people are more likely to choose options that lead to fairer outcomes, even if this may reduce their personal gains (Arrow, 1981; Samuelson, 1993; Sen, 1995).

The superior fairness judgments observed in older preschoolers (5–6 years) compared to younger preschoolers (3–4 years) can be attributed to the combined development of cognitive and social capacities. First, according to Piaget’s cognitive development theory, children aged 5–6 are transitioning from the preoperational stage to the concrete operational stage. During this period, children begin to overcome egocentric thinking and develop an understanding of concepts such as conservation and equivalence, which provide a cognitive foundation for forming fairness judgments. Additionally, older preschoolers exhibit significant advancements in theory of mind, enabling them to better understand others’ perspectives and needs (Wellman et al., 2011). This enhanced perspective-taking ability allows them to evaluate distribution scenarios more objectively, beyond self-interest.

Social cognitive development also plays a crucial role in older preschoolers’ fairness judgments. Research suggests that by the age of 5–6, children develop a deeper understanding of social norms and increasingly internalize equal distribution as a universally accepted standard of fairness (Hod-Shemer et al., 2018). This internalization of social rules likely drives their preference for equal sharing in resource allocation tasks, reflecting their alignment with fairness principles. Furthermore, the maturation of executive functions in older preschoolers supports their ability to inhibit egocentric tendencies and integrate multiple sources of information (Steinbeis and Over, 2017), enabling them to demonstrate greater consistency and rationality in fairness-related decisions.

### The moderating role of advisor familiarity: from cognition to complex behavioral transition

Despite their mature understanding of the fairness principle, our findings from Experiments 1 and 2 indicate that 5–6-year-old preschoolers’ sharing decisions are significantly influenced by the familiarity of the advisor. This further validates the social information processing theory, which states that young children’s internal perceptions of fairness are influenced by external information, which in turn influences their sharing decisions (Arsenio and Lemerise, 2004). Specifically, they are willing to heed suggestions from familiar advisors, including newly acquainted experimenter advisors, but do not blindly follow suggestions from completely unfamiliar stranger advisors.

This pattern suggests that children’s sharing decisions are not solely based on their fairness cognition but are significantly modulated by their trust in familiar advisors. Recent research indicates that children prioritize suggestions from familiar social relationships and authority figures in their decision-making processes (Liang et al., 2020). Familiar advisors, as important socialization agents, establish trust through daily interactions, lending their suggestions greater authority in children’s minds (Grocke et al., 2018). This leads children to adjust their behavior according to these suggestions, sometimes even overriding their internal fairness principles.

The differential response to familiar versus unfamiliar advisors aligns with contemporary understanding of children’s social information processing. When processing social information, children consider the source’s credibility and relevance (Zhang et al., 2019). With unfamiliar advisors, the absence of prior interaction and emotional connection leads children to rely more heavily on their own judgment in determining sharing behavior (Wörle and Paulus, 2018). This selective social learning process reflects children’s developing ability to discriminate between different sources of social influence.

### Consistency between fairness judgments and sharing behavior

Experiments 1 and 2 revealed a developmental shift in the consistency between children’s fairness judgments and sharing behavior. Older children (5–6 years) more frequently aligned their actions with their judgments, while younger children (3–4 years) showed greater variability. This suggests a developing ability to translate fairness understanding into consistent prosocial action.

Several developmental factors contribute to this increased consistency in older children. Improved executive function, particularly inhibitory control, allows them to better regulate impulses and align actions with internalized moral standards (Diamond, 2013). This enhanced self-regulation helps resist selfish impulses and prioritize fairness. Furthermore, more sophisticated emotional regulation enables older children to manage feelings of loss or reluctance during sharing, facilitating decisions consistent with their moral reasoning (Song et al., 2018). Developing metacognitive skills also contribute, allowing children to monitor and adjust their behavior to match their fairness judgments (Schneider et al., 2022).

The inconsistency observed in younger children reflects their ongoing development. They are still acquiring the self-regulation and emotional control necessary to align judgments with actions. Limited inhibitory control may lead them to prioritize self-interest, even when they understand fairness principles (Carlson et al., 2014). Additionally, less developed emotional regulation makes it harder to overcome possessiveness during sharing (Song et al., 2018). Working memory constraints may also hinder their ability to maintain focus on fairness while enacting sharing behaviors (Alloway and Alloway, 2010). These limitations contribute to the “moral judgment-behavior gap,” where children understand fairness but struggle to consistently apply it (Hod-Shemer et al., 2018).

### The emotional rewards of generosity: How “over-sharing” enhances positive emotions in young children

Results from Experiments 1 and 2 demonstrated that when children shared more than what they perceived as fair—that is, engaged in “over-sharing” as indicated by higher dissonance scores—their self-reported emotion scores increased significantly, suggesting more positive emotional experiences. This finding aligns with previous research documenting discrepancies between children’s actual behavior and their verbal (or moral judgment) expressions (Smith et al., 2013), and further confirms the presence of a “moral judgment–behavior gap” among preschoolers, where fairness judgments and actual sharing behavior do not always correspond (Smith et al., 2013).

Further analysis revealed that the association between over-sharing and positive emotions was more pronounced in children aged 5–6 than those aged 3–4, indicating that the emotional benefits of generous behavior increase with cognitive and socioemotional development. According to self-determination theory, autonomous prosocial acts yield intrinsic satisfaction (Ryan and Deci, 2024); neurobiological research has also shown that prosocial behavior activates reward circuits in the brain, generating a “warm glow” (Luo, 2018; Wang et al., 2019). Acts of generosity that go beyond fairness norms may further enhance children’s prosocial self-concept and social connectedness (Paulus and Moore, 2017).

These age differences may be attributed to older children’s more advanced emotional understanding, greater grasp of social norms, and developing metacognitive abilities which enable them to better appreciate and express the positive emotions associated with generous acts (In contrast, younger children’s emotional awareness and expressive capacity remain less mature) (Thompson, 2011; Tomasello, 2025), which may limit their experience and articulation of these emotional benefits.

## Limitations and future directions

This study investigated the influence of suggestion fairness and advisor familiarity on preschoolers’ sharing behavior, yielding several significant findings. Nevertheless, certain limitations should be acknowledged. First, the advisor role in our study was restricted to teachers, which may be confounded by the inherent authority of teachers in educational settings. Consequently, children’s responses might not purely reflect the effect of familiarity. Their compliance with teachers’ suggestions could simultaneously be influenced by both deference to authority and familiarity relationships, making it difficult to disentangle the unique contributions of these two factors. Second, our study did not examine the impact of other types of advisors (such as peers or friends) on children’s sharing behavior. These non-authority yet potentially familiar advisors might elicit different patterns of compliance.

Based on these limitations, future research could extend this line of inquiry in several ways. First, researchers could introduce more diverse advisor roles, such as peers, unfamiliar adults, and family members, to distinguish between the effects of authority and familiarity. Second, future studies could delve deeper into the interactive effects of multiple factors, including intimate relationships and authority, to explore how children weigh different social cues when making sharing decisions. Finally, employing diverse measurement methods, such as behavioral observation and physiological indicators, would enable a more comprehensive understanding of children’s cognitive, emotional, and behavioral responses during the sharing decision-making process.

## Conclusion

1. Despite a more mature understanding of fairness, 5–6-year-old children are more susceptible to adult suggestions regarding sharing than 3–4-year-olds.
2. Five- to six-year-old children are more receptive to suggestions from familiar advisors compared to unfamiliar advisors or strangers.
3. Greater consistency between fairness judgments and sharing behavior is observed in 5–6-year-olds, while 3–4-year-olds exhibit a larger gap between their understanding of fairness and their actions.
4. Children who “over-share” (share more than they deem fair) experience more positive emotions.

## Data Availability

The original contributions presented in the study are included in the article/Supplementary material, further inquiries can be directed to the corresponding author.
